# Qualitative healthcare worker survey: Retrospective cross-sectional case study on COVID-19 in the African context

**DOI:** 10.1016/j.amsu.2022.103918

**Published:** 2022-06-10

**Authors:** Allison Benjamin, Amir Sultan, Mirghani Yousif, Abdelmajeed Moussa, Ehab Fawzy Abdo, Johnstone Kayandabila, Kenneth Ssebambulidde, Lucy Ochola, Ifeorah Ijeoma, Nasreen Syeda Quadri, Jose Daniel Debes

**Affiliations:** a University of Minnesota, Department of Medicine, Minneapolis, MN, USA; b Addis Ababa University, Faculty, Department of Gastroenterology, Addis Ababa, Ethiopia; c University of Gezira, Faculty of Pharmacy, Department of Clinical Pharmacy & Pharmacy Practice, Gezira, Sudan; d Aswan University Hospital, Faculty of Medicine, Department of Gastroenterology, Aswan, Egypt; e Al-Rajhi University Liver Hospital, Assiut University, Department of Tropical Medicine and Gastroenterology, Assiut, Egypt; f Arusha Lutheran Medical Centre, Head of Critical Care Unit & Covid-19 Isolation Unit, Department of Medicine, Arusha, Tanzania; g Makerere University, Medical Officer, College of Health Sciences, Kampala, Uganda; h Institute for Primate Research, Faculty Researcher, Nairobi, Kenya; i University of Nigeria, Faculty of Health Sciences, Department of Virology, Nsukka, Nigeria; j Allina Health, Faculty, Department of Internal Medicine and Pediatrics, Minneapolis, MN, USA; k Hennepin Healthcare, Faculty, Department of Gastroenterology and Hepatology, Minneapolis, MN, USA; l Erasmus MC, University Medical Center Rotterdam, Department of Medicine, the Netherlands

**Keywords:** COVID-19, Pandemic, Health personnel, Surveys and questionnaires, Africa, Global health

## Abstract

**Background:**

Despite the presence of COVID-19 epidemiologic data in Africa, there are gaps in the understanding of healthcare workers’ concerns and fears early in the pandemic.

**Methods:**

A retrospective cross-sectional multi-country pan-African qualitative survey case study on the perceived effects of the COVID-19 pandemic on healthcare workers in the continent focused specifically on personal safety and misinformation. The survey was distributed to 13 countries via snowball sampling of practitioners between April 22 and May 15, 2020. The survey solicited free-form answers, resulting in a large spectrum of responses. Qualitative analysis included open and axial coding methods for thematic emergence.

**Results:**

A total of 489 analyzable responses were recorded. The majority of respondents (n = 273, 57%) highlighted personal safety concerns including lack of resources and training to prevent infection (33%); fear of infection and transmission (24%); lack of public awareness and compliance with regulations (12%); governmental concerns (9%) and economic insecurity (11%) amongst others. 328 respondents (67%) reported having heard misinformation about COVID-19. Responses included misinformation regarding origin of the virus (11%), false modes of transmission (6%), differential effect for specific groups (30%), unproven cures (35%), and disbelief in existence (11%). Responses for misinformation and fears revealed categorical associations between certain countries.

**Conclusion:**

Addressing fears and concerns of frontline healthcare workers facilitates their essential role in combating community misinformation, and further understanding could provide essential insight to institutions and governments to direct resource allotment and community education.

## Highlights

• African healthcare workers had personal safety concerns with the COVID-19 pandemic.
• Healthcare workers heard a variety of misinformation in their communities.
• Surveying healthcare workers contextualizes lived experience on the frontline.
• Surveying healthcare workers can identify resource needs and community education.

## 1 Introduction

As the COVID-19 pandemic affects life across the globe, the Africa Centers for Disease Control and Prevention has called to attention case numbers in Africa [[1], [2], [3]]. Although epidemiological data exists in the African region, less is known about healthcare workers' lived experience from a qualitative lens early in the pandemic. Summative cross-sectional survey results across several African countries are similarly limited. Several studies have focused on availability of resources; knowledge and attitude of frontline workers; and stressors on healthcare workers (HCW) [[4], [5], [6], [7]]. Understanding HCW experiences can inform region-wide initiatives to address areas of improvement in the current and future waves.

Here we describe a retrospective cross-sectional multi-country pan-African qualitative survey case study on the perceived effects of the COVID-19 pandemic on HCW in Africa. We first describe the methods of seeking qualitative data from HCW participants across the continent followed by results collating HCW input in their own words. The qualitative questions in this study surveyed HCW about personal safety concerns and misinformation about COVID-19. Thereafter, we explore the practical implications of the qualitative responses and emergent themes on current considerations across Africa in regards to managing the COVID-19 pandemic and its effects on HCW.

## 2 Methods

The methods were fully compliant with the STROCSS 2021 criteria [8]. This work was submitted to the Research Registry with UIN 7932 [9].

Design. The study design was a retrospective cross-sectional case study. We designed a 43-question survey seeking HCW perceptions about COVID-19. The questions surveyed were created and agreed upon via internal discussion of the authors in addition to input on clarity and feasibility from 10 additional partners working in Africa. Two questions were qualitative free-response prompts about personal safety and misinformation: a) “Do you have concerns about your personal safety due to the COVID-19 lockdown in your country? If yes, then why? If no, please write no; b) “Have you heard misinformation about COVID-19 circulating in the community? What specifically have you heard?”

Data collection. The Google Forms survey was distributed in a rapid fashion to remain relevant to the evolving context of COVID-19 in Africa. It was distributed to 13 countries through the African Hepatitis B Network (africanhepbnetwork.org) via snowball sampling of practitioners through email and WhatsApp. Responses were collected between April 22 and May 15, 2020. Participation in the entirety of the survey was voluntary and anonymous; as such, written consent was not requested by participants. The ethical clearance of the study was approved by Hennepin Healthcare Institutional Review Board. Further detailed information regarding the survey distribution and clinical data is described in our prior publications [6,7].

Analysis. The qualitative analysis used open and axial coding methods with Microsoft Excel. As such, researchers identified common themes, coded responses into those themes, and grouped themes into larger categories. Final categories, which contained a minimum of n = 20 responses, were derived from the data and named based on their constituents. Responses not fitting into a specific category were placed into a category labeled “other”. The personal safety question had six total categories; the misinformation question had seven. For the analysis of the misinformation question, categories were further subdivided into subcategories with a minimum of n = 5 responses. Many responses referenced multiple categories and thus were coded as such. A summary of emerging themes from qualitative analysis of both survey questions are noted in Table 1. Remarkable responses to the personal safety survey question were placed as direct quotes in Table 2.

**Table 1:** Emergent themes from qualitative analysis.

Do you have concerns about your personal safety due to COVID-19 lockdown in your country? If yes, then why?	Have you heard misinformation about COVID-19 circulating in the community? What specifically have you heard?
Lack of Resources and Training at Work to Prevent Infection (33%)	Origin (11%)
Fear of Infection/Transmission (24%)	False Modes of Transmission (6%)
Lack of Public Awareness and Compliance with Regulations (12%)	Severity (10%)
Governmental Concerns (9%)	Differential Effect (30%)
Economic Insecurity (11%)	Unproven Cures (35%)
Other (11%)	Disbelief in the existence of COVID-19 (11%)
	Case Reporting (7%)

**Table 2:** Personal safety respondent quotes.

Categories	Quotes
Lack of Resources	“We live in [the] third world where health facilities are scarce.”
	“The hospital is overwhelmed by patient[s].”
	“Hospital workers are not supplied by proper PPEs; imagine doctors being given cloth masks at outpatient clinics.”
	“We keep seeing [COVID-19] clients suspects and we currently have no capacity of testing them so we rather refer and manage symptomatically; we doubt if they really go for testing or they just go back home to their families and spread the disease even more.”
Fear of Infection/Transmission	“I'm exposed to a lot of people as a doctor on a daily basis so I'm highly susceptible to acquiring the virus, however, there is no PPE to make my day to day activities less risky.”
	“I cannot put myself in danger in an attempt to help others … it is only when I am alive [that I can] help my community.”
	“It's very difficult to know all the infected People due to limited testing capacity. One can get exposed [in] the process of treating even unknown Coronavirus cases.”
	“Too many unscreened people who could infect others”
	“It is hard to tell asymptomatic cases who are potential transmitters.”
	“Risk of catching Covid while traveling to work by bus”
	“The transportation system is a bottleneck in prevention.”
Lack of Public Awareness and Compliance with Regulations	“It seems like the lockdown is optional for some and not for all.”
	“I'm afraid we've not taken the pandemic seriously enough as [the] majority of the people are still not practicing social distancing, hand hygiene and other measures to stop the spread.”
	“The possibility of contacting a possible transmitter from the community scares me.”
	“Negligence is overwhelming; people don't act cautious for different reasons.”
	“The community is not aware of the magnitude and seriousness of the pandemic and measures of prevention”
	“I live in a society [with not] enough awareness and cooperation toward [the] prevention of covid 19.”
Governmental Concerns	“I'm underprotected. The fact that health care was a joke to my country existed before covid, but now I'm not just serving under brutal [conditions], helping while I needed help, underpaid, dishonest and fraud healthcare system, Now I'm about to die from it … and still, the theatrical act from [the] ministry of health and federal government continues, while me and my underprotected, underappreciated colleagues ([in] addition to millions of patients) will take the heavy fall … I see the worst coming, and I can't seem to have a say in it!”
	“The higher officials are highly ignorant about the health system. Rather they are focusing on false reports.”
Economic Insecurity	“Hunger is becoming worse than even the COVID-19 pandemic.” “Most Ugandans live ‘hand to mouth’ concerned about the poverty that will be caused by this.”
Other	“[I] am feeding [at] restaurants and hotels because raw materials for cooking at home are not easily accessible.”
	“Health workers aren't being paid and yet they are required to go to work during a lockdown when they don't have private cars.”
	“The use [of] Uber Taxi transport”
	“It is very expensive to use an office-hired car and also delays the time of reaching [the] office.”
	“People are stigmatizing health professionals.”
	“Isolation is causing stigma so patients are afraid to come to hospital for fear of acquiring the infection or forced to institutional isolation leaving their families without support.”
	“The enforcement of the lockdown … is being abused to include everyone not observing curfew. This has increased stigmatization on [the] covid pandemic since the infection is now more associated with criminal offense. Many patients with mild symptoms are now hiding [though] hospitals [are] recording low numbers.”

Categorization of responses determined observed associations between data and individual African countries. A stepwise approach to determine associations between categories and countries is described next. Proportional analysis of responses revealed percentages per country. This method was used to account for considerable variation in the number of survey respondents per country. If a proportion varied considerably from the rest in that category, it was further investigated to determine association. The Gambia (n = 1), South Sudan (n = 1), Malawi (n = 2), Rwanda (n = 1), and Sierra Leone (n = 2) were excluded from country analysis since there were too few responses to determine association.

## 3 Results

Demographics. We obtained 535 total responses between April 22nd and May 15th of 2020. Duplicate submissions and incomplete responses were removed resulting in 489 analyzable responses. Most respondents were from Ethiopia (51%), Tanzania (13%), Nigeria (9%), Egypt (7%), Sudan (5%), Uganda (5%), and Kenya (5%). The median age of respondents was 33 years (IQR 26–36), 73% of respondents were male, and the majority (63%) were physicians. Of physicians, 18% were consultants and 44% were trainees. Remaining respondents included pharmacists, nurses, students or clinical/medical officers.

### 3.1 Inquiries about personal safety

#### 3.1.1 Question: do you have concerns about your personal safety due to COVID-19 lockdown in your country? If yes, then why? If no, please write no

**Fig. 1:**
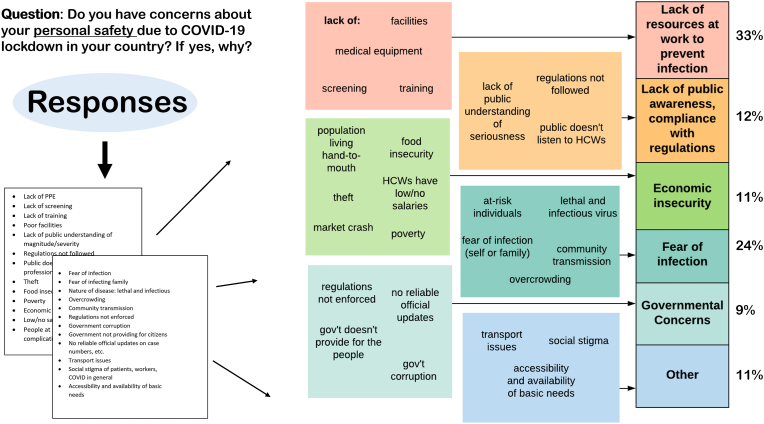
Personal safety responses.

This question solicited free-form answers; as such, we obtained a large spectrum of responses (Fig. 1). The majority of respondents (n = 273, 57%) indicated concerns about safety, 8% left this question blank, and 2% indicated not having a lockdown. Respondents’ concerns were categorized into the following areas: lack of resources and training at work to prevent infection (33%); fear of infection and transmission (24%); lack of public awareness and compliance with regulations (12%); governmental concerns (9%); economic insecurity (11%); and other (11%). Additional direct quotes from HCW are noted in Table 1.

#### 3.1.2 Lack of resources and training at work to prevent infection (33%)

Respondents were concerned about the ability of healthcare workers, systems, and facilities to handle the COVID-19 pandemic due to a lack of resources. They stated hospitals were “overwhelmed by patients” with inadequate supplies and medical equipment such as “[SARS-CoV-2] testing capacity,” “oxygen cylinders, concentrators, mechanical ventilators, N95s and surgical face masks, lab investigation (RT PCR), and drugs.” The most common concern was a lack of personal protective equipment (PPE), with some respondents reporting having “no PPE at all.”

Additionally, medical professionals voiced concern about a lack of proper training or protocol in place to successfully navigate the health crisis, noting “inadequate training on safety measure[s],” “compromised teaching with no leadership or guidance,” and “we don't apply [the] triage system perfectly.”

#### 3.1.3 Fear of infection/transmission (24%)

Due to the reported “infectious character,” “deadly” nature, and “rapid spread” of the novel coronavirus, participants expressed fear of becoming infected with SARS-CoV-2. Two HCWs attributed their concerns to having personal pre-existing health conditions. There were concerns about unsafe work environments—many respondents worried about contracting the virus due to the possibility of transmission to patients, families, and communities. Community transmission through overcrowding was a problem in respondents’ workplaces, roads, and public transportation.

#### 3.1.4 Lack of public awareness and compliance with regulations (12%)

Concerns in this category were related to variable public adherence with COVID-19 mitigation strategies. Respondents reported that many in their communities did not “abide by the lockdown,” “observe recommended precautionary measures,” or “adhere to ministry of health instructions.” Respondents attributed public noncompliance to a lack of understanding in their communities, detailing “the community is not aware of the magnitude and seriousness of the pandemic and measures of prevention.” One respondent ascribed “society … not strictly abiding by the lockdown” to “sociocultural habits [being] deep rooted,” and another commented “people are not willing to listen to the health care workers.”

#### 3.1.5 Governmental concerns (9%)

Survey participants expressed dissatisfaction with their governments’ responses to COVID-19 including a lack of reliable and regularly updated case reporting from governments. Respondents reported governmental responses were poorly-organized or nonexistent, describing “no coordinated effort to enforce … laws,” no “national restraint on movement of people,” and “poor … government policies.” Some were frustrated with a lack of government transparency, specifically “no daily updates on tests, number of cases or complications from the virus by the government.” Finally, a few respondents were concerned about the failure of their governments to provide supplies, shelter, and transportation to healthcare workers including basic needs and palliative care to the people.

#### 3.1.6 Economic insecurity (11%)

Primarily, respondents were concerned about poverty, since many in their countries lived “hand to mouth” and were prevented from working due to the pandemic. They also noted that theft and hunger were on the rise. Some respondents cited general economic instability, such as inflation or a possible market crash, as the root of their concern. Finally, three respondents highlighted HCW were being “underpaid” or not paid at all.

#### 3.1.7 Other (11%)

Respondents (n = 10) voiced concerns about transportation, such as HCW being “required to go to work during a lockdown when they don't have private cars … [and] aren't being paid.” HCW (n = 9) were also worried about the lack of accessibility and availability of essential resources such as food and supplies. Survey participants drew attention to social stigmatization of HCW, COVID-19 patients, and COVID-19 mitigation strategies.

### 3.2 Inquiries about misinformation

#### 3.2.1 Question: have you heard misinformation about COVID-19 circulating in the community? What specifically have you heard?

**Fig. 2:**
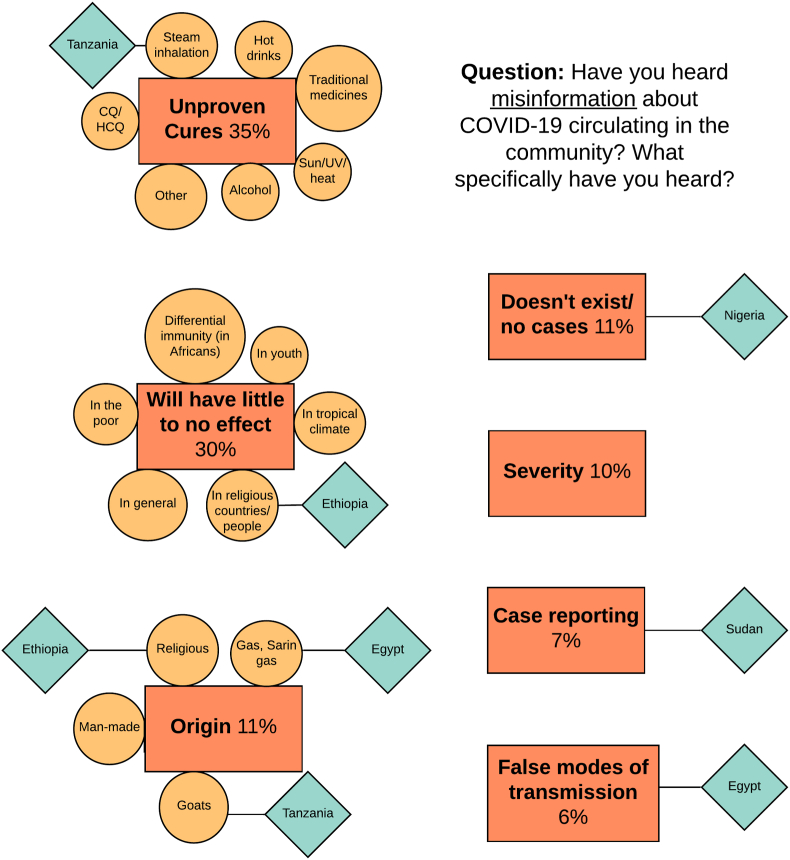
Misinformation responses.

The majority of respondents (n = 328, 67%) reported to have heard misinformation about COVID-19 and 16% left this question blank. Out of all respondents who indicated hearing misinformation, 89% (n = 293/328) provided specific example(s) of misinformation. Classification as misinformation was solely based on survey respondent categorization as such in response to this survey question, rather than factual accuracy. The categories of misinformation clustered around origin of the virus (11%), false modes of transmission (6%), severity (10%), differential effect (30%), unproven cures (35%), disbelief in existence (11%), and case reporting (7%). While there was much crossover between African countries in response categories to the personal safety question, responses to this misinformation question revealed associations with certain countries (Fig. 2).

### 3.3 Types of misinformation

#### 3.3.1 Origin (11%)

Respondents reported hearing misinformation that COVID-19 had a religious origin (n = 12; Ethiopia), had a man-made origin (n = 6), was sarin gas (n = 3; Egypt), or came from goats (n = 2; Tanzania).

#### 3.3.2 False modes of transmission (6%)

Respondents heard COVID-19 was transmitted “sexually,” by “mosquitoes,” by “animal carriers,” “over the phone,” and by “5G internet.” Four respondents believed “COVID-19 is airborne” to be misinformation. Remaining respondents confirmed misinformation about the mode of transmission of the virus but did not specify further.

#### 3.3.3 Severity (10%)

The great majority of respondents in this category (n = 23) reported misinformation that COVID-19 was not very serious, deadly, or contagious, or was similar to the common cold or flu.

#### 3.3.4 Differential effect (30%)

Many respondents (n = 34) described misinformation about SARS-CoV-2 having little to no effect on Africans, such as the disease not being “as severe or prevalent in blacks,” as “fatal as seen in other nations,” or not affecting Africans at all. One HCW heard “Africans are immune to COVID-19 based on previous exposure to diverse tropical infections.”

Respondents (n = 20) heard about a differential effect of COVID-19 in relation to climate: “The disease [has a] lower transmission rate in hot temperature[s],” and “The warm climate in our country prevents us from getting COVID.” Finally, respondents referred to SARS-CoV-2 having little to no effect in youth (n = 14) and religious countries or people (n = 11; Ethiopia).

#### 3.3.5 Unproven cures (35%)

Nearly half (n = 59/103) of the respondents in this category heard traditional medicines or natural remedies protected against or treated COVID-19. Common responses were garlic (n = 8; Ethiopia and n = 1; Nigeria), ginger (n = 5; Ethiopia and n = 1; Tanzania), lemon (n = 3; Ethiopia and n = 2; Kenya), and onion (n = 2; Ethiopia) as home remedies. Other subcategories of misinformation of cures or protective methods against COVID-19 were drinking alcohol (n = 12); hydroxychloroquine/chloroquine (n = 9); hot drinks such as warm water or tea (n = 7); sunlight, ultraviolet light, and/or heat (n = 7); and steam inhalation (n = 5; Tanzania and n = 1; Ethiopia).

#### 3.3.6 Disbelief in the existence of COVID-19 (11%)

This category was mainly associated with Nigeria (n = 12/32). Respondents stated many in their communities believed COVID-19 to be a “hoax,” “rumor,” “political conspiracy,” and “scam by [the] government to siphon public funds.” One respondent related hearing “the swabs being imported are tainted with the virus so we all test positive.” Because of disbelief in the existence of COVID-19, one respondent shared that community members “throw cautions to the wind.”

#### 3.3.7 Case reporting (7%)

This category was associated with Sudan. Many respondents believed there was a “false record of confirmed cases” and “misinformation about [the] number affected by COVID-19.” One Sudanese respondent stated, “in Sudan, we are in [a] transition period so many people get benefits from mobilizing [the] wrong information like increasing the number of new cases, number of deaths, etc.” A Tanzanian HCW shared “the president [is] giving false information to the nation regarding [the] statistics of COVID-19.” Other respondents noted a misperception in their communities that COVID-19 had disappeared due to actions that had been taken. Due to “reopening of transport, market, other activities, and fewer … daily positive case reports [from] the ministry,” a HCW said COVID-19 was “perceived as decreased/disappeared in our country.”

## 4 Discussion

Our qualitative survey uncovered variability in the lived experience of HCW across the African continent early in the COVID-19 pandemic. Notably, 57% of respondents confirmed concerns about their personal safety and 67% heard misinformation in their communities.

This data contributes to the current literature on HCW perceptions and experience of the COVID-19 pandemic. Several studies evaluated knowledge, attitude and practices in individual cross-sectional studies, for example, in Egypt, Nigeria, Uganda, Cameroon, South Africa, Ethiopia and Mozambique [[10], [11], [12], [13], [14], [15]]. The mental health impact on frontline workers included anxiety, depression, stress and poor sleep with resultant concern for burnout [[16], [17], [18]]. Indeed, a study from our group found a dramatic increase in feelings of depression and hopelessness among HCW [6,7]. These stressors not only impact HCW job satisfaction and retention of the frontline workforce, but also the ability of HCW to properly respond to medical needs during the pandemic [19,20]. Similarly, HCW have experienced stigmatization as a result of the COVID-19 pandemic [6,14]. Semo et al. astutely recommended increased mental health and psychosocial support across Africa including diversified methods of delivery to reach HCWs [21].

Our survey results identify societal needs as a core component of personal safety through its notable intersection with governmental public policies. The pandemic had a dramatic personal effect on HCWs, including lack of resources, social stigma, economic insecurity, fears of infection spreading, challenges with governmental policies, and community misinformation. Participants identified PPE, HCW compensation, and unified public health messaging and adherence as critical to personal safety.

Surveying misinformation is a key component to understanding the beliefs behind health behaviors and regional patterns of information dissemination. Roozenbeek et al. investigated COVID-19 misinformation across the UK, Ireland, USA, Spain and Mexico, finding a link between misinformation susceptibility, vaccine hesitancy and lower likelihood of complying with public health guidance [22]. Early in the COVID-19 pandemic while scientific studies were in process, there were few definitive studies to confirm the validity of claims made surrounding transmission and treatment [23,24]. Some of the misinformation shared by respondents was practice-based at the time the survey was conducted, for example the use of hydroxychloroquine as treatment [25]. HCW could survey misinformation in their communities to target public health messaging addressing commonly held beliefs and exploring their legitimacy as an intervention strategy in itself, applying qualitative methods.

Limitations. Our study had a number of limitations. First, although the survey included respondents from 13 African countries, each country was not equally represented in the data. More respondents from countries with lower representation would have provided comprehensive information for stronger associations. Demographically, respondents were largely male and majority physicians, which is not representative of the entire workforce across Africa. Rural areas were underrepresented in the data since respondents were largely residents of urban areas working in medical centers. Future studies would benefit from respondent pools with greater diversity in country, gender, health profession, political preference, and setting. A further challenge of qualitative analysis is a limited ability to capture the original intention of a respondent. It is possible that responses could have been misinterpreted or important feedback missed due to lack of clarity during the analysis. The survey was only distributed in English; this language barrier may have led to a misinterpretation of the data and limited the depth of the answers in the absence of respondents’ preferred language. Conducting future surveys in multiple languages would be mutually beneficial to respondents and researchers.

### 4.1 Conclusion

Qualitative research seeks to explore a group in their context and setting [26]. Institutions can combine efforts to unify region-specific resource allotment and community education. Periodic open-ended surveys directly seeking input from HCW globally can determine the most appropriate needs on the ground in a quickly shifting global context.

## Ethical approval

Hennepin Healthcare Institutional Review Board and Ethics Committee.

## Sources of funding

Funding was received through the 10.13039/100006090University of Minnesota Center for Global Health and Social Responsibility; 10.13039/100007249UMN COVID-19 Rapid Response Grants and 10.13039/100000867Robert Wood Johnson Foundation to Jose Daniel Debes.

## Author contribution

Allison Benjamin: Data analysis, Writing the Paper. Amir Sultan: Study concept, Data Collection, Writing the Paper. Mirghani Yousif: Study concept, Data Collection, Writing the Paper. Abdelmajeed Moussa: Study concept, Data Collection, Writing the Paper. Ehab Fawzy Abdo: Study concept, Data Collection, Writing the Paper. Johnstone Kayandabila: Study concept, Data Collection, Writing the Paper. Kenneth Ssebambulidde: Study concept, Data Collection, Writing the Paper.Lucy Ochola: Study concept, Data Collection, Writing the Paper. Ifeorah Ijeoma: Study concept, Data Collection, Writing the Paper. Nasreen Syeda Quadri: Study concept, Data Collection, Writing the Paper. Jose Daniel Debes: Securing funding, Study concept, Data Analysis, Data Collection, Writing the Paper.

## Registration of research studies

1. Name of the registry: N/A.

2. Unique Identifying number or registration ID: N/A.

3. Hyperlink to your specific registration (must be publicly accessible and will be checked): N/A.

## Guarantor

Jose D. Debes.

## Consent

Consent to participate:Participation in our anonymous survey was entirely voluntary; written consent was not requested by participants.

## Availability of data and material

The corresponding author may be contacted for all material used and data obtained from this study.

## Provenance and peer review

Not commissioned, externally peer-reviewed.

## Declaration of competing interest

N/A.
